# Case Report: A rare case of coronary artery anomaly (right coronary artery-pulmonary artery fistula with diffuse dilatation)

**DOI:** 10.3389/fcvm.2025.1664375

**Published:** 2025-08-11

**Authors:** Xiaoming Wang, Jie Yu, Changzheng Chen, Zhihua Yang, Lixia Yang

**Affiliations:** ^1^ Department of Cardiovascular Medicine, 920th Hospital of the Joint Logistics Support Force of the Chinese People's Liberation Army, Kunming, Yunnan, China; ^2^ Department of Cardiothoracic Surgery, 920th Hospital of the Joint Logistics Support Force of the Chinese People's Liberation Army, Kunming, Yunnan, China

**Keywords:** coronary artery anomalies, coronary aneurysm, coronary fistula, coronary angiography, heart failure coronary artery anomalies, heart failure

## Abstract

**Background:**

Congenital coronary artery anomalies (CAAs) are rare cardiovascular disorders that often present with non-specific symptoms, making diagnosis and treatment particularly challenging. These anomalies can occur in various forms, including anomalous origin of a coronary artery, coronary artery fistula, and coronary artery tortuosity, among others. The lack of specific symptoms often leads to delayed diagnosis, which can have significant implications for patient outcomes. In addition, the treatment options for CAAs vary depending on the type and severity of the anomaly, posing further therapeutic challenges for healthcare providers.

**Case presentation:**

This case report describes a 39-year-old male presenting with heart failure secondary to a giant right coronary artery-pulmonary artery fistula (RCA-PA fistula) accompanied by diffuse aneurysmal dilatation. The patient exhibited acute decompensated heart failure, atrial fibrillation, and severe left ventricular enlargement, initially misattributed to valvular disease. Coronary angiography and surgical exploration confirmed a tortuous, dilated RCA fistula draining into the pulmonary artery, with diffuse aneurysmal changes compromising myocardial perfusion. Due to the complexity of aneurysmal anatomy and high surgical risk, conservative management was adopted, leading to symptomatic improvement.

**Conclusions:**

This case highlights the need for heightened clinical suspicion of CAAs in young patients with atypical heart failure and emphasizes multidisciplinary strategies for managing complex coronary malformations.

## Introduction

Coronary artery anomalies (CAA) are relatively rare but clinically significant congenital heterogeneous structural defects in cardiology. In recent years, with the advancement of imaging techniques, the detection rate of coronary artery abnormalities has increased, reaching 0.21%–5.79% (1). Coronary artery fistula (CAFs) refers to abnormal connections between the coronary arteries and the heart lumen or blood vessels, accounting for 14% of all CAAs (2). Most of them do not exhibit signs, symptoms, or complications, so they may go unrecognized. On the other hand, some of them are associated with myocardial ischemia and related consequences, namely angina, infarction, arrhythmias, and sudden cardiac death. Early diagnosis poses a challenge.

Coronary-to-pulmonary artery fistulas (CPAFs) represent 28% of CAFs, but co-occurrence with diffuse aneurysmal dilatation is extremely uncommon (3). Hemodynamic abnormalities from large fistulas can induce coronary steal, leading to myocardial ischemia and ventricular dysfunction (4).

This report describes a 39-year-old male with heart failure secondary to a giant right coronary artery-pulmonary artery fistula (RCA-PA fistula) with diffuse aneurysmal changes. Initial evaluations misattributed symptoms to valvular disease, highlighting the diagnostic pitfalls in CAAs. The case underscores the need for heightened suspicion of complex coronary anomalies in young patients without typical atherosclerotic risk factors.

## Case presentation

A 39-year-old male was admitted to the hospital due to “repeated chest tightness and shortness of breath for 2 years, with aggravation lasting for half a month”. The patient has repeatedly experienced chest tightness and shortness of breath after activity since 2020. Since April 2022, the above symptoms have worsened, accompanied by paroxysmal nocturnal dyspnea. There is no edema in both lower extremities. An electrocardiogram was performed at the local county hospital, showing atrial fibrillation. An echocardiographic examination indicated anterior leaflet prolapse of the mitral valve with moderate regurgitation, as well as enlargement of the left atrium (LA: 58 mm), right atrium (RA: 54 mm), and left ventricle (LV: 73 mm). The left ventricular ejection fraction (EF) was measured at 41%, and the NT-proBNP level was significantly elevated at 9934 pg/ml. In 2010 (at the age of 29), due to “cerebral hemorrhage and cerebral infarction”, he developed hemiplegia in the left upper limb and experienced a decline in muscle strength in the left lower limb, with muscle strength grade 3. He denied any history of diabetes and reported a 20-year smoking history, consuming 10 cigarettes daily without cessation. Physical examination revealed a blood pressure of 103/75 mmHg, an enlarged cardiac silhouette extending to the lower left, a heart rate of 96 beats per minute with an irregular rhythm, and a grade 4/6 systolic rumbling murmur auscultated in the mitral valve area. The abdomen was soft without tenderness or rebound tenderness, and the liver and spleen were not palpable below the costal margin. There was no lower extremity edema, and pathological signs were negative. Upon admission, the ECG demonstrated atrial fibrillation with a rapid ventricular response and premature ventricular contractions.

**Figure 1:**
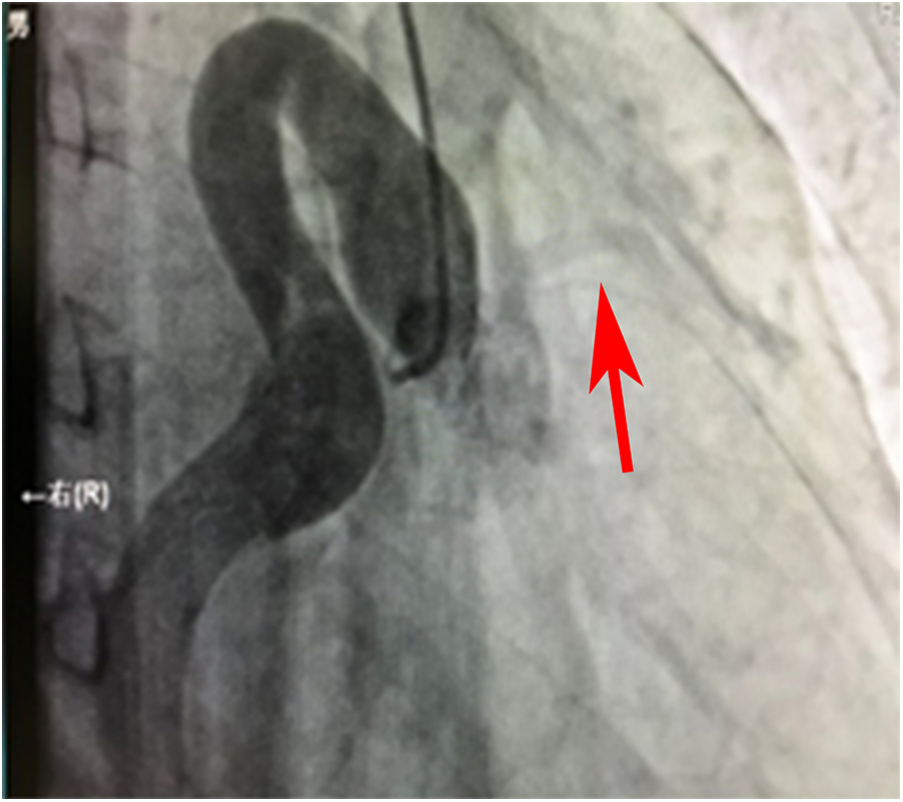
Angiographic image of the ostium and proximal segment of the RCA.

**Figure 2:**
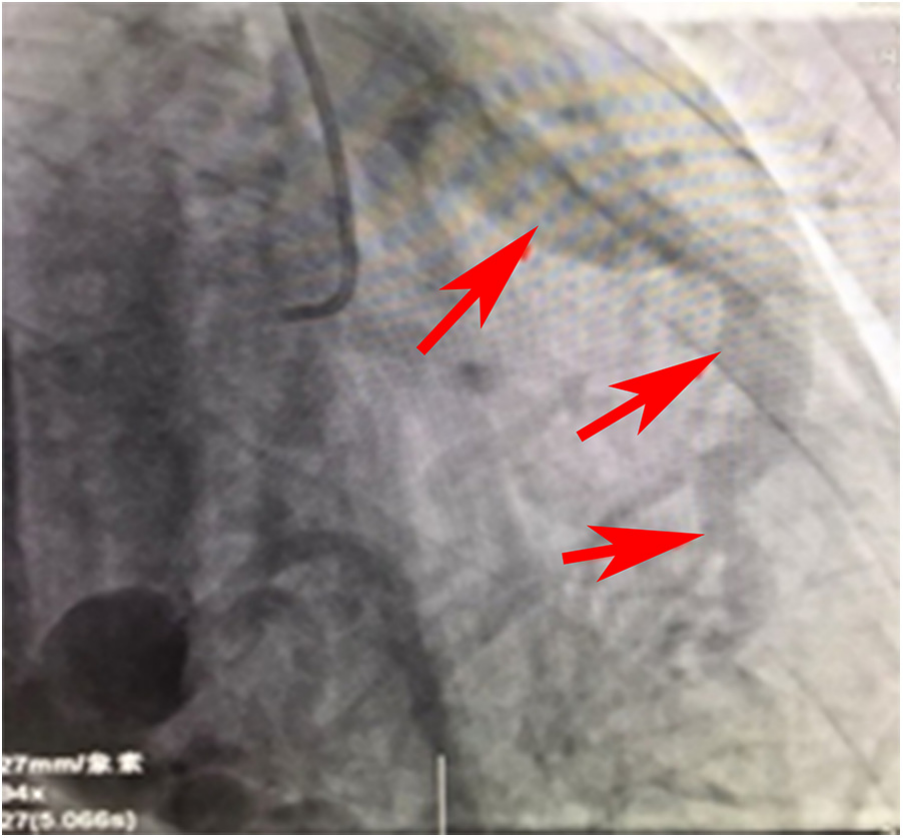
Angiographic image of the RCA draining into the pulmonary artery.

**Figure 3:**
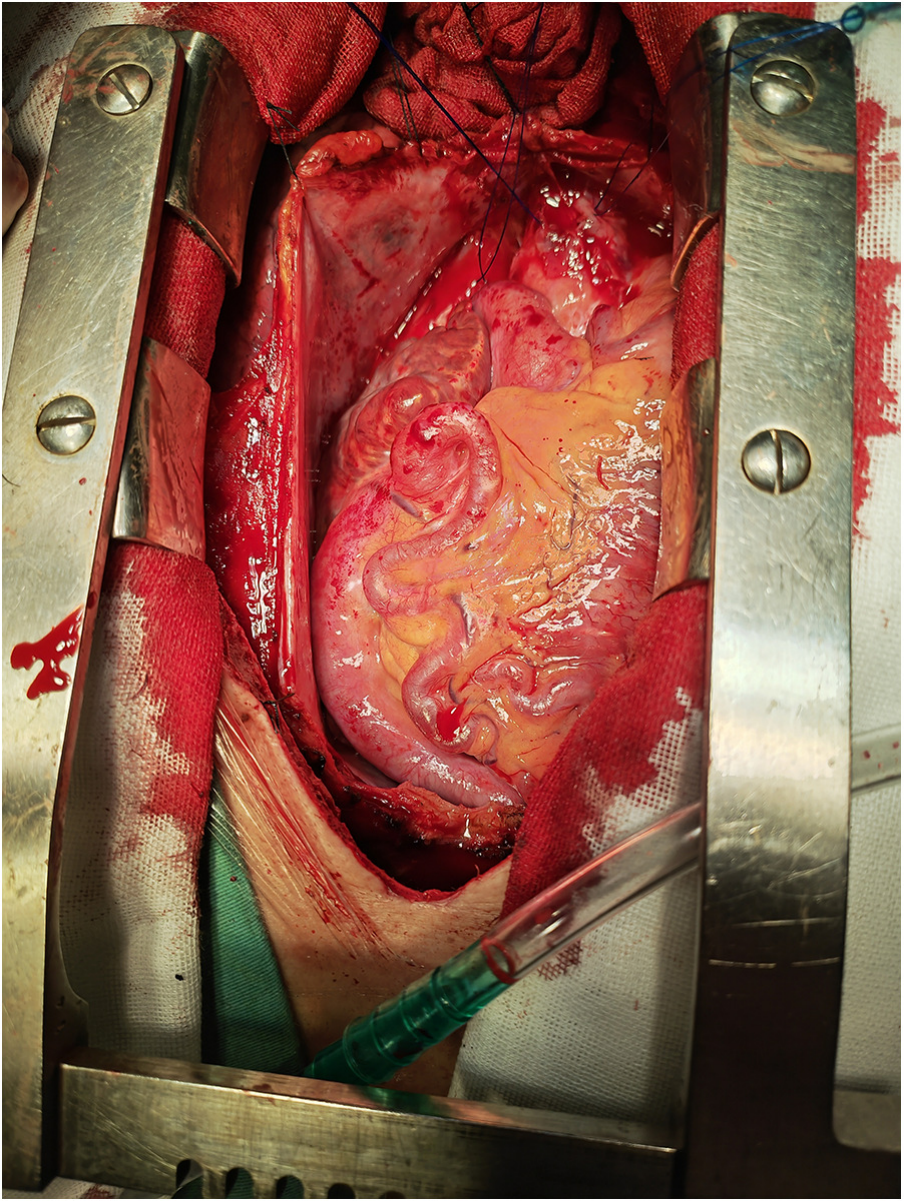
Overall view of abnormal coronary arteries under direct vision.

**Figure 4:**
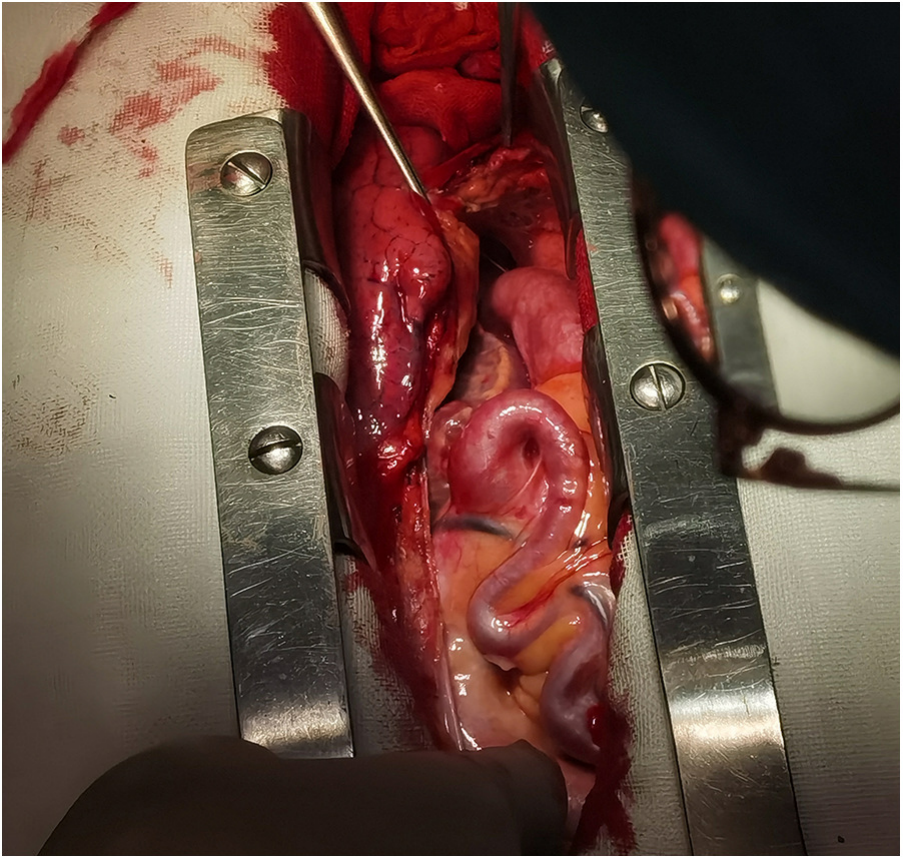
Local view of abnormal coronary arteries under direct vision.

The initial diagnoses included acute exacerbation of chronic heart failure, mitral valve prolapse with insufficiency, atrial fibrillation, coronary atherosclerotic heart disease, and sequelae of cerebral hemorrhage. Subsequent echocardiography at our institution revealed an irregular rhythm with a relatively rapid heart rate of 131 beats per minute, further enlargement of the left atrium (LA: 66 mm) and left ventricle (LV: 77 mm), mitral valve anterior leaflet prolapse with associated mitral valve insufficiency, a general reduction in left ventricular wall amplitude by 2 to 3 mm, severe pulmonary hypertension (estimated pulmonary artery systolic pressure of approximately 61 mmHg based on tricuspid regurgitation), a small amount of pericardial effusion, and impaired left ventricular diastolic and systolic function (EF: 43%). Color Doppler flow imaging demonstrated significant mitral regurgitation, moderate tricuspid regurgitation, and a small degree of aortic regurgitation. Coronary angiography was performed at an elective date, and the results are illustrated in Figures 1, 2, the video data of coronary angiography can be found in the Supplementary Materials. The patient was subsequently transferred to the cardiac surgery department for mitral valve repair. During surgical exploration, notable cardiac enlargement was observed, with normal development of the aorta and pulmonary trunk. Both the left atrium and left ventricle were enlarged, and the right coronary artery is notably thick and large in its entirety, with tortuosity and swelling, accompanied by a palpable thrill (Figures 3, 4). It was concluded that the abnormal development of the coronary artery resulted in insufficient myocardial blood supply and decreased myocardial contractility. The valve replacement surgery was anticipated to encounter challenges related to cardiac resuscitation and inadequate coronary perfusion. After thorough discussions with the patient's family, a decision was made to abort the surgical intervention. The patient then received treatment for heart failure, resulting in symptom improvement, and was subsequently discharged.

Figure 1 shows that the right coronary artery opens into the aorta and is cystic throughout the entire process. A small left coronary artery can be seen on the left side (red arrow).

Figure 2 is a continuous animation of the angiography in Figure 1. The angiography shows that the right coronary artery communicates with the pulmonary artery and flows into it. The red arrow indicates that the blood flows into the pulmonary artery.

Figures 3, 4 shows abnormally dilated coronary arteries after thoracotomy in cardiac surgery.

## Discussion

The estimated prevalence of CAAs varies significantly, ranging from 0.21 to 5.79% (1). CAAs can be classified as CAAs of origin (anomalous pulmonary origin of the coronaries, anomalous aortic origin of the coronaries, congenital atresia of the left main artery), of course (myocardial or coronary bridging, coronary aneurysm), and of termination (coronary arteriovenous fistula, coronary stenosis) (5). The clinical presentation of CAAs often demonstrates nonspecific features, encompassing a spectrum from asymptomatic status or mild complaints (e.g., angina pectoris or dyspnea) to life-threatening manifestations including sudden cardiac death, thereby complicating early diagnostic efforts (6, 7). We herein report a 39-year-old male who presented with acute-onset heart failure concomitant with newly diagnosed atrial fibrillation. Initial differential diagnoses centered on dilated cardiomyopathy and rheumatic valvular heart disease, whereas intrinsic coronary structural anomalies remained undetected until comprehensive imaging evaluations. This diagnostic trajectory underscores the necessity for heightened clinical suspicion toward congenital coronary anomalies in young patients with heart failure devoid of traditional atherosclerotic risk profiles.

The rapid advancement of contemporary imaging modalities has revolutionized the diagnostic precision of CAAs. While conventional coronary angiography historically served as the gold standard, its diagnostic limitations—including invasiveness, relatively low spatial resolution, and the absence of three-dimensional reconstructions—have precipitated its gradual replacement by coronary computed tomography angiography (CCTA). CCTA has emerged as the first-line imaging modality for CAAs characterization due to its non-invasive nature, exceptional spatial resolution, and three-dimensional reconstruction capabilities (8). Auxiliary techniques including transthoracic echocardiography (TTE), transesophageal echocardiography (TEE), and contrast-enhanced CT/MRI provide complementary hemodynamic and structural insights, particularly in delineating fistula flow dynamics and aneurysmal wall integrity (9). In this case, we have confirmed the diagnosis by coronary angiography. Although no further coronary CT angiography (CCTA) was performed, we directly demonstrated a clear image of the right coronary artery -pulmonary artery fistula (RCA-PA) with a diffuse giant aneurysm through surgical open-heart surgery. These valuable data provide important imaging data for the study of complex coronary malformations, greatly deepening the understanding and understanding of this condition.

Coronary-to-pulmonary artery fistulas (CPAFs) account for approximately 28% of congenital coronary artery fistulas (CAFs) (3), however, their coexistence with giant diffuse aneurysmal dilatation remains exceedingly rare. Hemodynamically, chronic shunting from the high-pressure coronary system to the low-resistance pulmonary circulation induces coronary steal phenomena, which typically remain asymptomatic in patients with small fistula orifices. In contrast, large-caliber fistulas may manifest with exertional dyspnea, fatigue, palpitations, angina pectoris, or even congestive heart failure (4). Termination of coronary arteries in low-pressure chambers subjects the vessel wall to persistent abnormal shear stress, driving progressive vascular dilatation, tortuosity, and mural degeneration—key antecedents to aneurysmal transformation and rupture risk (10). Notably, massive aneurysms may exacerbate ventricular volume overload, myocardial ischemia (via embolism or steal physiology), and valvular dysfunction due to mechanical compression (11). The clinical course of this case vividly demonstrates the close connection between progressive left ventricular enlargement and mitral regurgitation and the pathological mechanism. The uniqueness of the case lies in the fact that the huge diffuse tumor-like dilation accompanying the coronary artery fistula presents a significantly widened and distorted shape, in sharp contrast to the common regular morphology of cystic or fusiform aneurysms. This “massive diffuse dilation” subjects the vascular wall to “abnormal shear stress”. Due to the large size and distorted shape of the lumen, the direction of blood flow is disordered. The continuous high shear stress not only aggravates the degeneration of the vascular wall (such as the rupture of the elastic fibers in the media and the proliferation of smooth muscle), but also further promotes the progression of the aneurysm. A vicious cycle of “dilation—increased shear stress—further dilation” has been formed, which seriously affects the normal function of blood vessels. Progressive left ventricular enlargement combined with massive mitral regurgitation directly confirms the pathological mechanism that “aneurysms exacerbate ventricular volume overload and mechanical compression leads to valve dysfunction”. This phenomenon is relatively rare in common CAFs, presenting more severe and specific myocardial and valve involvement. The unique value of this case lies not only in its “rare pathological combination”, but also in the fact that its clinical course directly reveals the intrinsic connection between coronary hemodynamic changes (shunt, shear stress) and myocardial remodeling (left ventricular enlargement) as well as valve function (mitral regurgitation). This complete chain from abnormal blood flow to organ damage provides irreplaceable clinical evidence for understanding the pathophysiological mechanism when CAFs is combined with aneurysms, and has significant research value.

Congenital coronary artery aneurysm represent an exceptionally rare clinical entity, whereas acquired coronary artery aneurysm are secondary to diverse systemic pathologies, including atherosclerosis, syphilitic vasculitis, and Kawasaki disease (12). Kawasaki disease is the most common type of vasculitis associated with acquired CAAs, with approximately 80% of patients under 5 years of age (13). Untreated acute-phase Kawasaki disease [e.g., lack of intravenous immunoglobulin (IVIG) therapy] is an established independent risk factor for major adverse cardiac events (MACEs), as residual coronary lesions may progress to severe complications (e.g., aneurysmal rupture, thrombosis, or stenosis) in adulthood (14). Through detailed medical history collection, it was found that the patient's parents reported that he had experienced high fever (the specific body temperature was not recorded), generalized rashes and diarrhea at one month of age after birth. The local health institution provided antipyretic and antibiotic treatment as “pneumonia” (the specific drugs and course of treatment are unknown). This history of fever with rash in infancy is highly consistent with the clinical manifestations of Kawasaki disease (fever lasting ≥5 days, with skin and mucous membrane damage as the core manifestation) (15). However, due to the limitations of early medical conditions, Kawasaki disease was missed and the treatment with gamma globulin was delayed, which became an important pathological basis for subsequent coronary artery lesions.

The patient first experienced a cerebrovascular event (cerebral infarction and cerebral hemorrhage) at the age of 29, which prompted clinical attention to potential systemic vascular lesions. Based on the analysis of the natural history, uncontrolled Kawasaki disease in infancy triggers chronic inflammatory responses in the coronary arteries, leading to vascular endothelial dysfunction and medium smooth muscle injury, which gradually develops into coronary aneurysmal dilation. The huge diffuse coronary aneurysmal dilation shown in this case is actually the result of the combined effect of Kawasaki disease-related vascular remodeling (manifested as full-thickness thickening of the vascular wall and lumen tumor-like protrusion) and congenital CPAFs dynamic abnormalities (continuous shunting of the high-pressure coronary artery system to the low-pressure pulmonary circulation): the former lays the foundation for vascular structural lesions, while the latter intensifies the tumor-like dilation process through abnormal shear stress (mechanical damage to the vascular wall caused by continuous high-speed shunt), forming a pathological cascade reaction of “inflammatory damage—hemodynamic load—vascular remodeling”.

It is worth noting that during the 28-year clinical silent period before the age of 29, the patient did not present with typical prodromal symptoms such as angina pectoris and heart failure, suggesting that the compensatory dilation of the giant coronary aneurysm may mask progressive myocardial ischemia and cardiac function impairment. It is not until after the occurrence of cerebrovascular events that complex vascular lesions are revealed through imaging examinations, but by then the opportunity for early intervention (such as coronary artery fistula occlusion, aneurysm reconstruction, etc.) has been missed. Follow-up data showed that the patient died of multiple organ failure induced by severe heart failure in June 2023. The course of the disease from the first appearance of heart failure symptoms to the end of life was approximately 3 years. For complex coronary artery lesions discovered in adulthood, especially in patients with congenital CAFs, it is necessary to trace the history of fever in infancy and early childhood, and comprehensively evaluate the interaction between secondary factors of vascular lesions (such as inflammatory vascular disease) and congenital factors. Formulate individualized imaging follow-up plans (it is recommended to perform coronary CTA or cardiovascular MRI annually to assess tumor changes) and intervention strategies (including interventional embolization, surgical reconstruction or heart failure device treatment). The progression of the disease course in this case suggests that even during decades of clinical silence, untreated Kawasaki disease-related vascular lesions can still synergistically promote fatal cardiovascular events in conjunction with congenital heart malformations, emphasizing the significance of establishing a full life-cycle vascular lesion monitoring system for improving the prognosis of such patients.

Therapeutic management of CAAs necessitates tailored strategies based on lesion complexity and associated complications. Major complications include angina pectoris, myocardial infarction, sudden cardiac death, thrombosis, thromboembolism, arteriovenous fistula formation, vasospasm, and vessel rupture (16). The treatment strategy should be individualized considering the anatomic characteristics and the risk of complications. For some simple coronary artery fistula, interventional treatment can be considered. For complex coronary origin malformations, surgery may be a better option, and the American College of Cardiology/American Heart Association recommends percutaneous or surgical closure of large fistulas (17). Common surgical methods include resection or ligation of the proximal and distal aneurysms and coronary artery bypass grafting. Previous reports by Kinsing Ko (18) and Tang W (19) demonstrated favorable outcomes following combined fistula closure, giant aneurysm resection, and CABG in patients with right coronary artery (RCA) aneurysms. In this case, due to aneurysm involvement of the right main coronary artery and severe tortuosity, fistula closure combined with aneurysm resection plus coronary artery bypass grafting (CABG) could be theoretically adopted. However, due to the complexity of tumor anatomy and the technical requirements of myocardial protection during operation, conservative treatment was finally adopted. This treatment dilemma highlights the importance of establishing multidisciplinary cardiac teams in the diagnosis and treatment of complex coronary artery malformations, and the need to further explore individualized treatment options in the future.

## Conclusion

As a rare congenital cardiovascular malformation, the diagnosis and treatment of CAAs are extremely complicated and challenging. The reported case presented the imaging features of RCA-PA fistula complicated with diffuse giant aneurysm for the first time, providing a valuable reference for future clinical practice. It is suggested that suspected cases should be screened by coronary CT angiography (CCTA) as early as possible in clinical application, and a multidisciplinary collaborative diagnosis and treatment model should be established to further optimize the prognosis of patients.

## Data Availability

The original contributions presented in the study are included in the article/Supplementary Material, further inquiries can be directed to the corresponding author.
